# Unmasking Refractory Hypothyroidism: Persistent Iron-Levothyroxine Interaction Despite Appropriate Dose Separation

**DOI:** 10.7759/cureus.112442

**Published:** 2026-07-11

**Authors:** Zalatt Pann Ei, Thaw Tar Soe, Kaung Htet Kyaw, Hsu Wai Phyo, Nyan Tun Lin

**Affiliations:** 1 Internal Medicine, Lister Hospital, East and North Hertfordshire Teaching NHS Trust, Stevenage, GBR; 2 Endocrinology and Diabetes, Lister Hospital, East and North Hertfordshire Teaching NHS Trust, Stevenage, GBR

**Keywords:** drug-induced malabsorption, iron-thyroxine interaction, levothyroxine absorption, refractory hypothyroidism, thyroxine absorption test

## Abstract

Refractory hypothyroidism despite high-dose levothyroxine therapy is most commonly attributed to poor adherence or gastrointestinal malabsorption; however, drug-drug interactions remain an important and frequently under-recognised cause. Oral iron is known to impair levothyroxine absorption through chelation, and current clinical guidance recommends dose separation to mitigate this effect.

We report the case of a 44-year-old woman with severe biochemical hypothyroidism, with thyroid-stimulating hormone (TSH) >100 mU/L (reference range: 0.27-4.2 mU/L), free thyroxine (FT4) 2.4 pmol/L (reference range: 11-22 pmol/L), and free triiodothyronine (FT3) 1.4 pmol/L (reference range: 3.5-6.5 pmol/L), despite reported adherence to escalating doses of levothyroxine up to 250 µg daily over nine months. She was concurrently taking oral ferrous sulphate 200 mg once nightly for iron deficiency anaemia, while levothyroxine was taken first thing in the morning, ensuring a separation interval of at least four hours in accordance with standard recommendations.

A supervised levothyroxine absorption test performed according to the Manchester University NHS Foundation Trust Levothyroxine Absorption Test Protocol demonstrated adequate gastrointestinal absorption of levothyroxine, effectively excluding true malabsorption. Following discontinuation of oral iron and administration of intravenous iron, there was rapid biochemical improvement, with normalisation of TSH to 0.42 mU/L within six weeks.

This case highlights that a clinically significant iron-levothyroxine interaction may persist despite appropriate dose separation. Clinicians should maintain a high index of suspicion for pharmacological interference in patients with refractory hypothyroidism.

## Introduction

Hypothyroidism is a common endocrine disorder. The prevalence of spontaneous hypothyroidism ranges between 1% and 2% in the UK [1,2]. In the United Kingdom, epidemiological data suggest a similar burden, with higher prevalence observed in women and increasing age groups [1]. Levothyroxine (L-thyroxine) remains the standard first-line therapy and is highly effective in restoring biochemical euthyroidism [1]. Despite its efficacy, a subset of patients exhibits persistent hypothyroidism despite apparently adequate or even supraphysiological dosing. This condition, often referred to as refractory hypothyroidism, may arise due to a range of factors, including non-adherence, gastrointestinal disorders, and pharmacological interference [3,4].

In clinical practice, treatment failure is frequently multifactorial. Observational studies have demonstrated that levothyroxine performance is highly sensitive to administration practices, including timing of ingestion and co-administration with other agents [5]. Similarly, audit-based data highlight that variations in administration technique can lead to clinically significant differences in thyroid function control [6].

Drug-drug interactions represent a major contributor to impaired levothyroxine efficacy. Agents such as iron, calcium, and proton pump inhibitors are well recognised to interfere with levothyroxine absorption [7]. Levothyroxine absorption is further influenced by gastrointestinal and formulation-related factors, including gastric acidity, intestinal transit, and excipient composition [8]. Recent studies suggest that levothyroxine absorption varies between individuals, meaning that standard mitigation strategies, including dose separation, may not be universally effective [9]. Clinical reports have described persistent hypothyroidism despite adherence to recommended administration strategies, emphasising the complexity of levothyroxine pharmacokinetics in real-world settings [10]. In addition, lactose intolerance has been identified as a potential contributor to impaired levothyroxine absorption, with evidence demonstrating improved biochemical control following transition to lactose-free or liquid formulations [11].

Here, we present a case of severe refractory hypothyroidism in which clinically significant iron-levothyroxine interaction persisted despite appropriate dose separation, highlighting an important limitation of current management strategies.

## Case presentation

A 44-year-old woman with established primary hypothyroidism was admitted with severe biochemical hypothyroidism, with thyroid-stimulating hormone (TSH) >100 mU/L (reference range: 0.27-4.2 mU/L) and free thyroxine (FT4) 2.4 pmol/L (reference range: 11-22 pmol/L), despite reliable adherence according to the patient as well as the medication records from her general practitioner (GP). Over nine months, her levothyroxine dose had been progressively escalated from 150 µg daily (1.6 µg/kg/day; body weight 90 kg) to 175 µg, then 200 µg over six months, followed by 225 µg after four months, ultimately reaching 250 µg daily without biochemical improvement.

She reported fatigue, progressive weight gain, and severe menorrhagia. Her medical history included human immunodeficiency virus infection managed with long-acting rilpivirine injections, seronegative inflammatory arthritis treated with sulfasalazine, lactose intolerance, and iron deficiency anaemia treated with oral iron supplementation. She was not using any contraception. She reported taking oral iron sulphate 200 mg once a day every evening, which was at least four hours apart from levothyroxine in accordance with current clinical recommendations.

Given persistent hypothyroidism despite high-dose therapy, gastrointestinal malabsorption was initially suspected. A structured evaluation for malabsorptive disorders was undertaken. Coeliac serology was negative, and faecal elastase levels were within the normal range, making coeliac disease and pancreatic exocrine insufficiency unlikely. These findings further supported the absence of an underlying gastrointestinal malabsorptive disorder.

**Figure 1:**
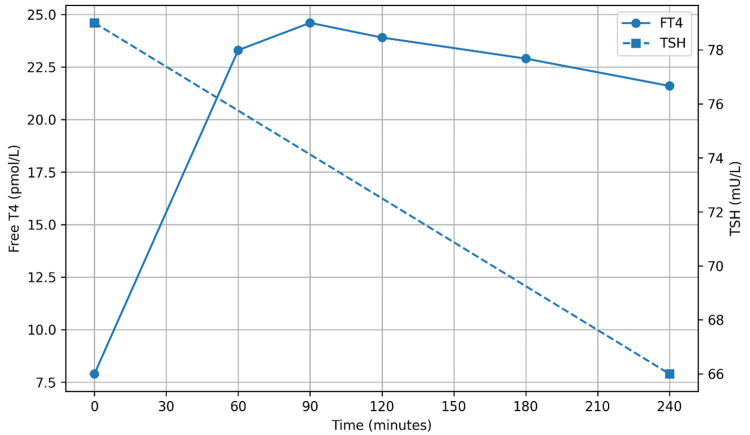
Levothyroxine absorption test demonstrating adequate gastrointestinal absorption. Serial free thyroxine (FT4) measurements following administration of 1000 µg oral levothyroxine show a rise of greater than 50% from baseline, consistent with adequate levothyroxine absorption. Thyroid-stimulating hormone (TSH) levels are shown at baseline and 240 minutes for reference.

A supervised levothyroxine absorption test (1000 µg orally) was performed following temporary discontinuation of oral iron. Serial measurements demonstrated a marked rise in FT4 from 7.9 pmol/L at baseline to 23.9 pmol/L at 120 minutes and 21.6 pmol/L at 240 minutes, representing an increase of greater than 50% from baseline. According to the Manchester University NHS Foundation Trust Levothyroxine Absorption Test Protocol, this constitutes a satisfactory biochemical response and confirms adequate gastrointestinal levothyroxine absorption (Figure 1) [12].

Further endocrine evaluation demonstrated normal follicle-stimulating hormone and luteinising hormone levels, elevated prolactin of 820 mU/L (reference range: 102-496 mU/L), and a low random cortisol level of 187 nmol/L. Repeat cannulated prolactin normalised to 455 mU/L, consistent with hypothyroidism-induced hyperprolactinaemia. A short Synacthen test subsequently confirmed adequate adrenal reserve, with cortisol levels rising from a baseline of 271 nmol/L to 599 nmol/L at 30 minutes and 704 nmol/L at 60 minutes.

In the absence of biochemical or clinical evidence of malabsorption, pharmacological interference from oral iron supplementation was considered the predominant contributor to treatment failure. Oral iron was discontinued and replaced with intravenous ferric carboxymaltose. Repeat thyroid function testing at six weeks demonstrated marked biochemical improvement, with normalisation of TSH to 0.42 mU/L. Verbal informed consent was obtained from the patient for publication of this case report and accompanying clinical information.

## Discussion

This case highlights an important and under-recognised limitation in the management of hypothyroidism: clinically significant drug interactions may persist despite adherence to recommended mitigation strategies.

Iron-levothyroxine interaction is well established and is primarily mediated through the formation of insoluble complexes within the gastrointestinal tract, reducing levothyroxine bioavailability [7,3]. Current American Thyroid Association guidance recommends separating administration by at least four hours to minimise this effect [1].

However, our case demonstrates that this strategy may not be universally effective. Despite appropriate dose separation and reported adherence, the patient remained profoundly hypothyroid until oral iron was discontinued. The rapid biochemical improvement following intravenous iron administration strongly supports persistent pharmacological interference as the possible primary mechanism.

Recent observational studies demonstrate that levothyroxine efficacy is highly sensitive to administration practices and co-administered medications [5,6]. Emerging pharmacokinetic evidence further highlights the complexity of levothyroxine absorption, including the influence of gastric pH, intestinal transit, and formulation characteristics [9].

The use of a supervised levothyroxine absorption test was critical in this case, allowing differentiation between true malabsorption and impaired absorption due to external factors. Negative coeliac serology and normal faecal elastase levels helped exclude common causes of gastrointestinal malabsorption, further supporting pharmacological interference as the primary mechanism [4]. Similar cases have been reported in the literature, reporting persistent hypothyroidism despite adherence and appropriate dose separation strategies [10].

In addition, formulation-related factors should be considered in refractory hypothyroidism. Levothyroxine tablets contain excipients such as lactose, which may affect absorption in susceptible individuals. Alternative formulations, including liquid or lactose-free preparations, may improve absorption in selected patients [8,11]. However, in this case, the marked improvement following withdrawal of oral iron suggests that drug interaction was the dominant mechanism. From a practical perspective, intravenous iron may represent a useful strategy in selected patients requiring iron replacement while receiving levothyroxine therapy, although oral iron remains first-line due to lower cost, accessibility, and established safety.

Future research is needed to better characterise the variability of iron-levothyroxine interactions among individual patients. Prospective pharmacokinetic studies could help determine whether the currently recommended four-hour separation interval is adequate for all patients or whether personalised administration strategies are required. Further evaluation of alternative levothyroxine formulations, including liquid and soft-gel preparations, may identify approaches that reduce susceptibility to drug interactions. In addition, studies comparing oral and intravenous iron replacement in patients requiring concomitant levothyroxine therapy may help inform future clinical guidelines.

## Conclusions

Clinically significant iron-levothyroxine interaction may persist despite appropriate dose separation. Refractory hypothyroidism should prompt careful evaluation for pharmacological interference, even in patients reporting good adherence. Levothyroxine absorption testing is a valuable diagnostic tool that can guide targeted and effective management.
